# An optogenetic system to control membrane phospholipid asymmetry through flippase activation in budding yeast

**DOI:** 10.1038/s41598-020-69459-0

**Published:** 2020-07-27

**Authors:** Tomomi Suzuki, Tetsuo Mioka, Kazuma Tanaka, Akira Nagatani

**Affiliations:** 1 Department of Botany, Graduate School of Science, Kyoto University, Oiwake-cho, Kitashirakawa, Sakyo-ku, Kyoto, 606-8502 Japan; 2 PRESTO, Japan Science and Technology Agency, Kawaguchi, Saitama 332-0012 Japan; 3 Division of Molecular Interaction, Institute for Genetic Medicine, Hokkaido University Graduate School of Life Science, Kita-ku, Sapporo, 060-0815 Japan

**Keywords:** Biological techniques, Cell biology, Molecular biology

## Abstract

Lipid asymmetry in biological membranes is essential for various cell functions, such as cell polarity, cytokinesis, and apoptosis. P4-ATPases (flippases) are involved in the generation of such asymmetry. In *Saccharomyces cerevisiae*, the protein kinases Fpk1p/Fpk2p activate the P4-ATPases Dnf1p/Dnf2p by phosphorylation. Previously, we have shown that a blue-light-dependent protein kinase, phototropin from *Chlamydomonas reinhardtii* (*Cr*PHOT), complements defects in an *fpk1*Δ *fpk2*Δ mutant. Herein, we investigated whether *Cr*PHOT optically regulates P4-ATPase activity. First, we demonstrated that the translocation of NBD-labelled phospholipids to the cytoplasmic leaflet via P4-ATPases was promoted by blue-light irradiation in *fpk1*Δ *fpk2*Δ cells with *Cr*PHOT. In addition, blue light completely suppressed the defects in membrane functions (such as endocytic recycling, actin depolarization, and apical-isotropic growth switching) caused by *fpk1*Δ *fpk2*Δ mutations. All responses required the kinase activity of *Cr*PHOT. Hence, these results indicate the utility of *Cr*PHOT as a powerful and first tool for optogenetic manipulation of P4-ATPase activity.

## Introduction

In eukaryotic cells, lipid bilayers exhibit asymmetric phospholipid distributions. This is particularly evident in the plasma membrane: the cytoplasmic leaflet has an abundance of phosphatidylserine (PS), phosphatidylethanolamine (PE), phosphatidylinositol (PI) and its derivatives, whereas the exoplasmic leaflets are rich in phosphatidylcholine (PC), sphingomyelin (SM) and glycosphingolipids^1,2^. These lipid asymmetries across membranes play a crucial role in many cell functions, such as vesicular transport^3^, cytokinesis^4,5^, cell signalling^6^, apoptosis^7^, cell migration^8^, and the immune response^9^. The asymmetry of phospholipids in biological membranes is established and maintained by lipid transporters^10^. A family of such transporters is P4-ATPase (i.e., flippase), which transports specific lipids from the exoplasmic leaflet to the cytoplasmic leaflet using the energy of ATP hydrolysis^10^. Most P4-ATPases interact with a noncatalytic subunit, Cdc50 family protein, for proper subcellular localization and ATPase activity^11^.

The P4-ATPase subfamily is widely conserved in eukaryotes^12^. To date, many studies in mammals, *Caenorhabditis elegans*, *Saccharomyces cerevisiae*, *Arabidopsis thaliana* and others have been reported^11,13,14,15^. As an example, mammalian studies have reported that knockdown of a P4-ATPase ATP8A1 disrupts the asymmetric distribution of PS in recycling endosomes (REs) and suppresses vesicle transport from endosomes^16^. The involvement of P4-ATPases in vesicular transport has been reported in various organisms and is considered to be a general function of P4-ATPases^3,17^. Probes that specifically bind to PS (e.g. LactC2-GFP^18^ and GFP-evt2-2XPH^19^) enable direct observation of the PS distribution in the cytosolic leaflet^16^. Analysis using a biotinylated PE probe (Ro09-0198 ^20^) in yeast cells showed that PE is specifically exposed on the surface of polarized ends such as the bud site and bud neck^21^. Mutation of P4-ATPase genes disrupts these PE polarized distributions in yeast cells^21^. As described above, genetic analysis using mutants and overexpression reveals many physiological functions of P4-ATPases in various organisms. Cell biological analysis using specific lipid probes reveals the location of intracellular phospholipids. In contrast, many issues regarding the spatiotemporal regulation of P4-ATPases and translocated phospholipids remain. To elucidate them, the development of a new tool is required.

The budding yeast *S. cerevisiae,* has five P4-ATPases, Drs2p, Dnf1p, Dnf2p, Dnf3p, and Neo1p. Neo1p is essential, while the others are redundantly essential for viability^22^. Dnf1p and Dnf2p have 69% amino acid sequence identity and are localized in the plasma membrane and inner membranes (trans-Golgi network (TGN), early endosomes and transport vesicles)^22,23,24,25^. Dnf1p/Dnf2p transport PE, PC, their lyso-forms, and monosaccharide glycosphingolipids like glucosylceramide (GlcCer) and galactosylceramide (GalCer). GlcCer is primarily transported by Dnf2p^13,23,26,27,28^. Both Dnf1p and Dnf2p require interaction with Lem3p, a member of the Cdc50p family, for subcellular localization and function; hence, *lem3*∆ and *dnf1*∆ *dnf2*∆ are phenocopies^29^. In both *dnf1*∆ *dnf2*∆ and *lem3*∆ mutants at a late mitotic phase, translocation of PE to the inner leaflet, actin depolarization at the bud tip and switching from apical to isotropic growth are significantly delayed compared to those in wild-type cells^23,30,31,32^. These results indicate that Dnf1p/Dnf2p-Lem3p are involved in the maintenance of cell polarity.

Dnf1p/Dnf2p-Lem3p are also involved in endocytic recycling of the vesicle-soluble *N*-ethylmaleimide-sensitive factor attachment protein receptor (v-SNARE) Snc1p. Mutation of another P4-ATPase, Drs2p, or its partner subunit Cdc50p causes an internal accumulation of Snc1p, and a combination of *dnf1*∆ *dnf2*∆ (or *lem3*∆) and *drs2*∆ (or *cdc50*∆) mutations results in severe vesicle-transport abnormalities^22,29,32,33^. Hence, Dnf1p/Dnf2p-Lem3p and Drs2p-Cdc50p are redundant in the regulation of vesicle transport in this pathway.

The only factors regulating the activity of Dnf1p/Dnf2p are the Ser/Thr protein kinase Fpk1p and its paralog, Fpk2p. The *FPK1* gene was identified as a gene responsible for a synthetic lethal mutation with *cdc50*∆, and the *fpk1*Δ *fpk2*∆ mutant shows almost the same phenotypes as the *dnf1*Δ *dnf2*Δ or the *lem3*Δ mutant^32^. Moreover, Fpk1p phosphorylates Dnf1p/Dnf2p in vivo and *in vitro*^32,34,35,36^; thus, Fpk1p/Fpk2p are Dnf1p/Dnf2p-activating kinases. Fpk1p and Fpk2p share the highest sequence homology in yeast with the AGCVIII kinase domain of phototropins (PHOTs), plant specific blue-light (BL) photoreceptors^32,37^. We have previously introduced a PHOT from *Chlamydomonas reinhardtii* (*Cr*PHOT) into an *fpk1*Δ *fpk2*Δ mutant with a conditional Cdc50p mutant (*P*_*GAL1*_*-CDC50 fpk1*Δ *fpk2*Δ. As a result, we have shown that *Cr*PHOT complements the growth defect of the *fpk1*Δ *fpk2*Δ mutant in a BL-dependent manner^37^. This result indicates the possibility that *Cr*PHOT controls P4-ATPases in a light-dependent manner. Therefore, we examined whether this system could be used as a new optogenetic technology.

In this study, we reintroduced *Cr*PHOT into the *fpk1*Δ *fpk2*Δ mutant or the *P*_*GAL1*_*-CDC50 fpk1*Δ *fpk2*Δ mutant and biochemically assessed the lipid translocation activity to show that it was indeed regulated by BL irradiation. Furthermore, Dnf1p/Dnf2p-dependent cellular processes, such as vesicle transport, were regulated in these cells by BL. Our results suggest the potential of *Cr*PHOT as an optogenetic tool to regulate membrane functions.

## Results

### Optical control of yeast cell growth by Chlamydomonas PHOT

**Figure 1:**
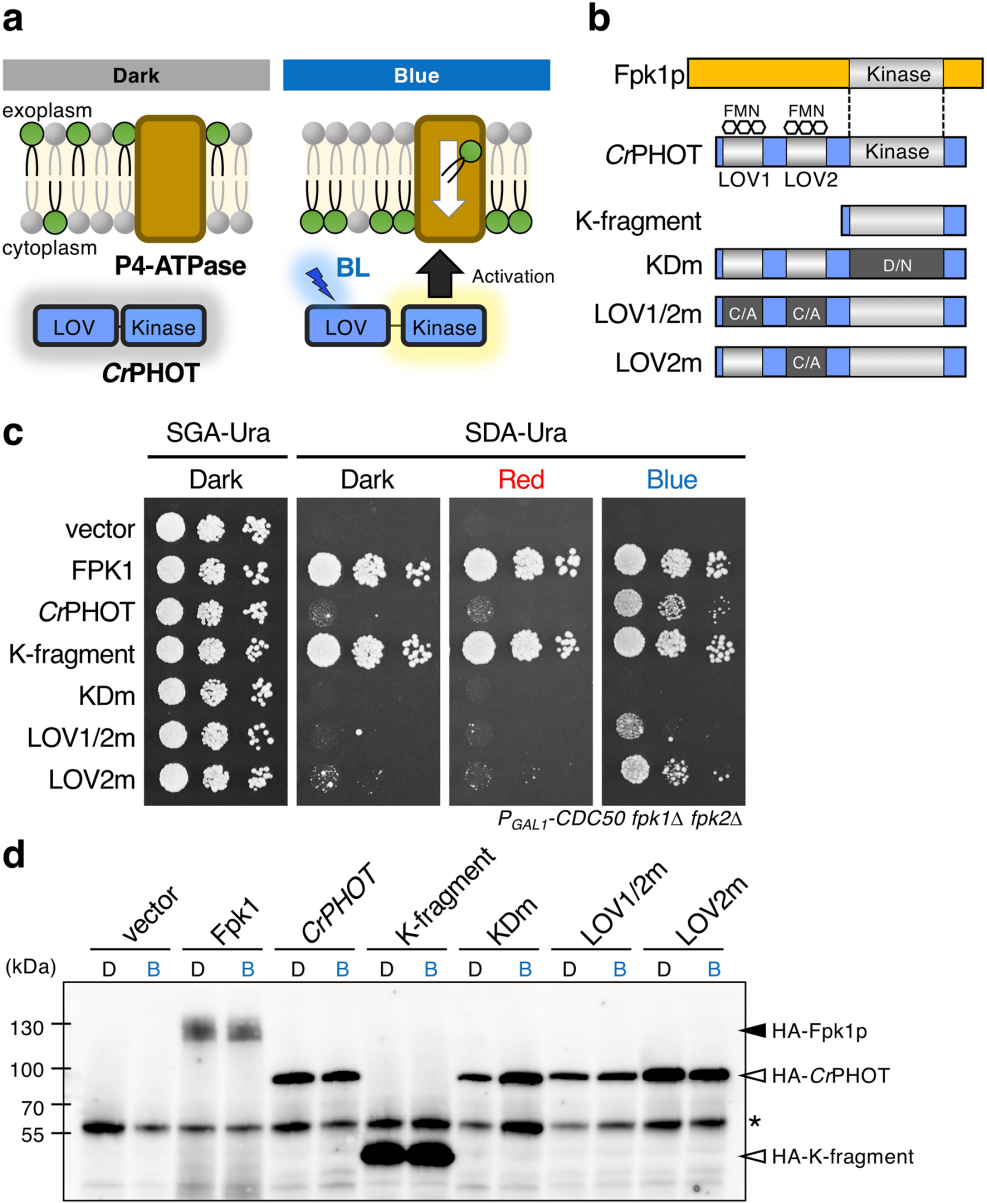
Light control of yeast cell growth by *Cr*PHOT. (**a**) A schematic of BL-induced activation of flippases through *Cr*PHOT in yeast. (**b**) Schematic illustration of Fpk1p and *Cr*PHOT and its derivatives. K-fragment, kinase domain fragment; KDm, kinase-dead mutant with D549N; LOV1/2 m and LOV2m, mutants with a decreased extent of light activation by substitution of conserved Cys in LOV1 and LOV2 domain or LOV2 domain only, respectively, to Ala. Flavin mononucleotide (FMN) is a chromophore of phototropin. (**c**) Growth of the yeast conditional mutant *P*_*GAL1*_*-CDC50 fpk1*Δ *fpk2*Δ carrying pRS416-*Cr*PHOT or its derivatives. Yeast cells were serially diluted and spotted onto plates containing galactose (SGA-Ura) or glucose (SDA-Ura), which were incubated in darkness (Dark) or under 10 μmol m^−2^ s^−1^ RL (Red) or BL (Blue) irradiation at 28 °C for 3 days. The experiments were performed more than three times with different biological samples. A representative image is shown in (**c**). (**d**) The protein level of *Cr*PHOT and its derivatives is not affected by light conditions. HA-fused Fpk1p and *Cr*PHOT and its derivatives were extracted from the yeast transformants (same strains shown in **c**) cultured in darkness (**d**) or under 10 μmol m^−2^ s^−1^ BL irradiation (**b**) at 28 °C for 16 h, which were then resolved by SDS-PAGE and analysed by immunoblotting with anti-HA antibodies. Asterisk indicates non-specific bands.

To establish a system to control P4-ATPase (flippase) activity by light (Fig. 1a), we attempted to prove that *Cr*PHOT regulates P4-ATPases at the molecular level. On the basis of the highest sequence homology among the kinase domains of Fpk1p/Fpk2p and *Cr*PHOT^32^, we have shown that *Cr*PHOT complements the synthetic lethality of a *P*_*GAL1*_*-CDC50 fpk1*Δ *fpk2*Δ yeast mutant in a BL-dependent manner^37^. In this study, we introduced *Cr*PHOT and its derivatives (Fig. 1b) into the mutant and examined its growth activity again. Consequently, we reconfirmed that *Cr*PHOT suppresses the growth defect of the *P*_*GAL1*_*-CDC50 fpk1*Δ *fpk2*Δ yeast mutant in glucose-containing medium under BL but not under red light (RL) or in darkness (Fig. 1c, *Cr*PHOT). Furthermore, a K-fragment with constitutive kinase activity suppressed the growth defect regardless of the light condition, and KDm with a kinase-dead mutation, failed to restore the growth even under BL (Fig. 1b, c)^37^. The substitution of a conserved cysteine (Cys) to an alanine (Ala) in the light-oxygen-voltage (LOV) domain is known to decrease the extent of light activation^38^. LOV2m, which has this mutation in the LOV2 domain, was used as a mutant to reduce light sensitivity in previous research^37^. LOV2m certainly reduced but did not completely abolish the ability to restore growth under BL^37^ (Fig. 1b, c). Therefore, we newly constructed LOV1/2 m, which has Cys to Ala mutations in two LOV domains (LOV1 and LOV2; Fig. 1b). The growth activity of LOV1/2 m under BL was considerably lower than that of LOV2m (Fig. 1c). Hence, LOV1/2 m was used as a mutant to reduce photosensitivity instead of LOV2m in this study.

We then examined the expression level of *Cr*PHOT and derived proteins in yeast with an anti-HA tag antibody (Fig. 1d). The level of *Cr*PHOT protein was not altered with light conditions as in previous analysis^37^. The amount of each *Cr*PHOT derivative was also not changed by BL irradiation, and their levels differed little. These results reconfirmed that complementation by *Cr*PHOT depends on its photoactivation and furthermore indicate that the degree of growth activity by the derivatives depends on their biochemical properties rather than the protein amount.

### CrPHOT and Dnf1p/Dnf2p P4-ATPases are localized in similar subcellular compartments

**Figure 2:**
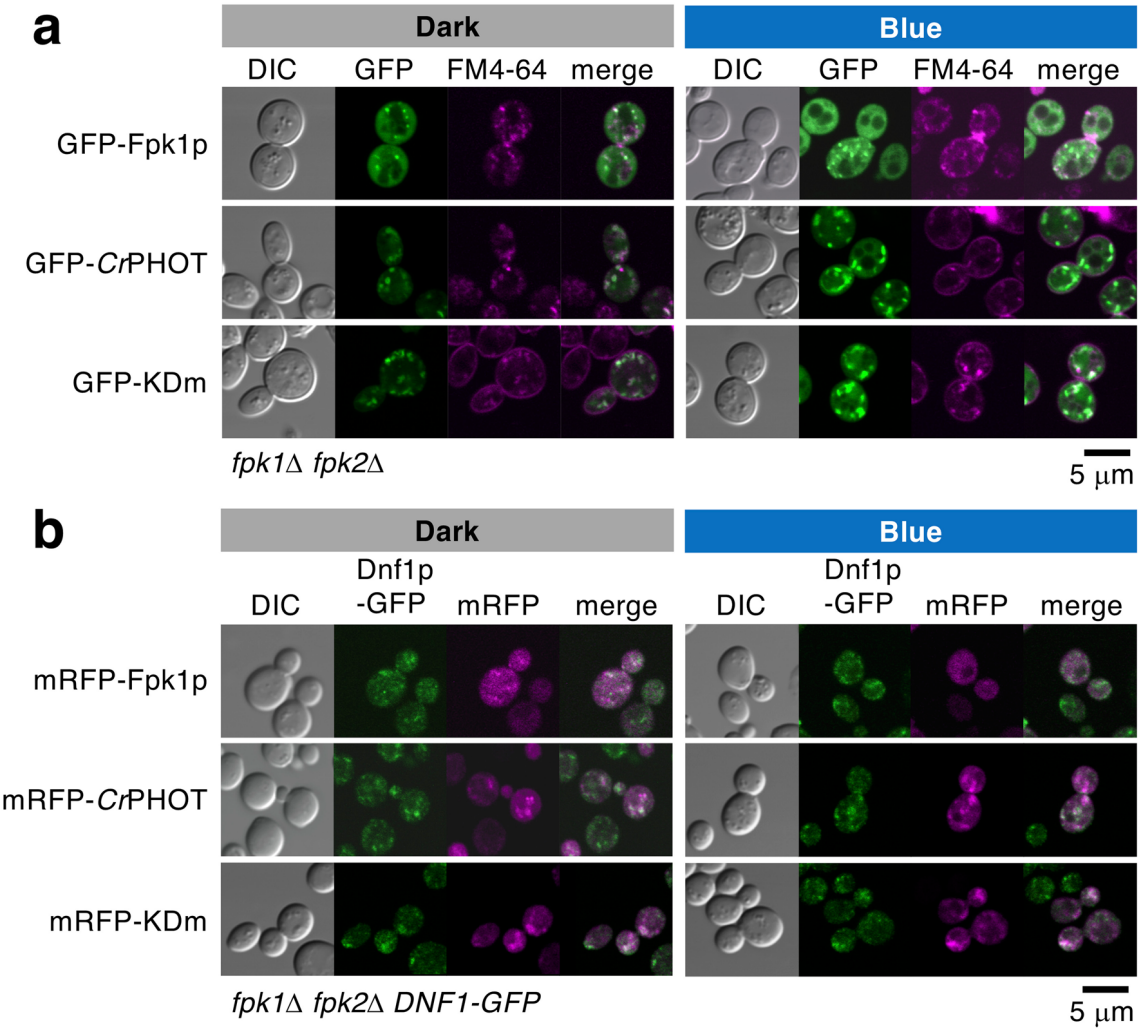
Intracellular localization of *Cr*PHOT is similar to that of FPK1p regardless of BL irradiation. (**a**) Punctate structures of GFP-*Cr*PHOT and GFP-KDm stained with FM4-64 were similar to that of GFP-FPK1p. Yeast *fpk1*Δ *fpk2*Δ cells carrying pRS416-GFP-FPK1, -GFP-*Cr*PHOT or -GFP-KDm were incubated at 25 °C with FM4-64 under each light condition, followed by confocal microscopic observation. (**b**) Confocal images of KKT332 (*fpk1*Δ *fpk2*Δ *DNF1-GFP*) cells carrying pKT1639 (pRS416-mRFP-FPK1), pRS416-mRFP-*Cr*PHOT or -mRFP-KDm. In (**a**) and (**b**), yeast cells were grown to logarithmic phase in darkness (Dark) or under 10 μmol m^−2^ s^−1^ BL irradiation (Blue) at 18 °C. Images were merged to compare the two signal patterns. Scale bars = 5 μm.

Fpk1p/Fpk2p, which regulate P4-ATPase activity, are mainly distributed throughout the cytoplasm but are partially localized in the early endosome/TGN and plasma membrane^25,32,39^. To clarify the control of P4-ATPases by *Cr*PHOT, we compared the intracellular localization of fluorescent protein-tagged *Cr*PHOT with that of Fpk1p. Before analysis, we confirmed that the tagged proteins were functional by a growth assay of the *P*_*GAL1*_*-CDC50 fpk1*Δ *fpk2*Δ mutant (Supplementary Fig. S1). An *fpk1*Δ *fpk2*Δ mutant expressing GFP-Fpk1p or GFP-*Cr*PHOT cultured in darkness was briefly stained with a lipophilic dye, FM4-64^32,40^, which was employed as a marker for the plasma membrane and endosomal/TGN compartments (Fig. 2a). GFP-*Cr*PHOT was occasionally observed at the plasma membrane but primarily localized to intracellular punctate structures, which mostly merged with the fluorescence of FM4-64 (64.4% of the GFP-*Cr*PHOT speckles were merged, n = 163; Fig. 2a). The localization was similar to that of GFP-Fpk1p (66.0% of the GFP-Fpk1p speckles were merged, n = 152; 67.5% in previous analysis^32^; Fig. 2a). The speckles of GFP-*Cr*PHOT, as with GFP-Fpk1p, were colocalized with another TGN marker, Sec7p-mRFP^32^ (Supplementary Fig. S2). These results suggest that GFP-*Cr*PHOT is primarily localized to endosomal/TGN compartments in yeast cells.

We then examined the effect of BL on the subcellular localization of GFP-*Cr*PHOT. This was because BL irradiation changes phototropin intracellular localization in plants^41,42^. The localization analysis by FM4-64 staining in yeast cells was performed under BL irradiation. The results showed that the localization of GFP-*Cr*PHOT under BL was the same as that in the dark and that most of its speckles merged with FM4-64 (62.5%, n = 120; Fig. 2a). To investigate the effect of kinase activity on *Cr*PHOT localization, we observed the localization of GFP-KDm. The localization pattern of GFP-KDm was the same as that of GFP-*Cr*PHOT regardless of the light condition (68.0% in the dark and 57.9% under BL, n = 166 and n = 164, respectively; Fig. 2a). These results suggest that the localization of *Cr*PHOT in yeast cells is not dependent on either BL or its own kinase activity.

Under normal culture conditions, Dnf1p and Dnf2p are primarily localized to early endosomal/TGN compartments and partially to the plasma membrane of the bud and bud neck^22,23,25,29,33^. Fpk1p is known to colocalize with Dnf1p/Dnf2p at endosomal/TGN compartments and the plasma membrane^32^. We then examined the colocalization of mRFP-*Cr*PHOT and Dnf1p-GFP in yeast cells. The results showed that Dnf1p-GFP was localized to punctate structures distributed throughout the cell, and many but not all of them colocalized with mRFP-*Cr*PHOT (Fig. 2b). The influence of BL or the introduction of mRFP-KDm on the localization of Dnf1p-GFP was hardly observed (Fig. 2b). The same results were found for the localization of Dnf2p-GFP (Supplementary Fig. S2). These results suggest that *Cr*PHOT, as is the case with Fpk1p, constitutively colocalizes with Dnf1p/Dnf2p in endosomal/TGN compartments.

### Optical control of phospholipid translocation by CrPHOT in a kinase activity-dependent manner

At the plasma membrane, Dnf1p/Dnf2p are involved in flipping phospholipids (mainly PC and PE) and glycosphingolipids^13,21,23,26,28,29,30^. Fpk1p/Fpk2p regulates phospholipid uptake through Dnf1p/Dnf2p across the plasma membrane^32,34,39,43^. Since *Cr*PHOT was able to complement Fpk1p/Fpk2p function in yeast cell growth^37^ (Fig. 1b), BL irradiation likely activates *Cr*PHOT to control phospholipid uptake in the same way as Fpk1p/Fpk2p.

**Figure 3:**
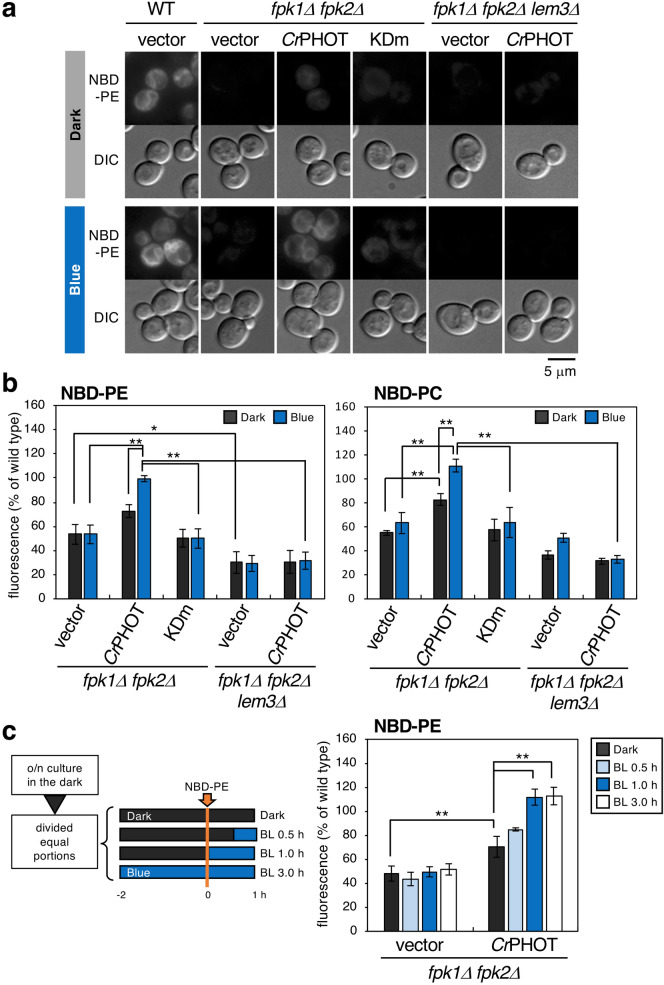
*Cr*PHOT promotes NBD-phospholipid flipping by flippases in a BL-dependent manner. (**a**) NBD-PE internalization by *Cr*PHOT under BL. Wild type (WT) harbouring vector plasmid, *fpk1*Δ *fpk2*Δ mutant harbouring vector, *Cr*PHOT or KDm plasmids, and KKT274 (*fpk1*Δ *fpk2*Δ *lem3*Δ) harbouring vector or *Cr*PHOT plasmids were grown in SC medium in darkness or under 10 μmol m^−2^ s^−1^ BL at 30 °C and treated with NBD-PE. A representative cell image obtained by microscopic observation is shown. Scale bar = 5 μm. (**b**) Quantification of internalized NBD-labelled phospholipids. Cells grown in SDA-U medium in the dark at 30 °C were labelled with NBD-PE or -PC for 60 min in the dark or under 10 μmol m^−2^ s^−1^ BL, and then washed with SD containing 2.5% BSA before flow cytometry. (**c**) Time course of NBD-labelled PE internalization by BL. Cells grown in the dark were incubated for a total of 3 h in the indicated light condition (dark, BL 0.5 h, BL 1.0 h, or BL 3.0 h), in which the last 1 h was incubated with the NBD-PE. The internalized NBD-phospholipids were quantitated by flow cytometry. Data are presented as the average percentage ± SD relative to wild-type measurements of three independent experiments (10,000 cells per sample) in each light condition (*p < 0.05, **p < 0.01; Tukey’s test).

We thus examined whether *Cr*PHOT controls the uptake of nitrobenzoxadiazole (NBD)-labelled phospholipids (NBD-PE and NBD-PC) in the *fpk1*Δ *fpk2*Δ mutant. NBD-phospholipids taken up by P4-ATPases are transported mainly to the endoplasmic reticulum (ER) in a short time, and strong fluorescence at the ER membrane is observed^30,44^. When NBD-PE was added, fluorescence was observed at the ER membrane in wild-type cells but not in the *fpk1*Δ *fpk2*Δ mutant (Fig. 3a, WT + vector and *fpk1*Δ *fpk2*Δ + vector). The internalized NBD-PE was quantified by flow cytometry of the cells (Fig. 3b). When the *fpk1*Δ *fpk2*Δ mutant harboured empty vector, the amount of NBD-PE was significantly decreased as described previously^32^ (53 ± 8% in the dark and 53 ± 8% under BL relative to wild-type levels, Fig. 3b). When the *fpk1*Δ *fpk2*Δ mutant harboured *Cr*PHOT plasmids, intracellular fluorescence was hardly observed and showed a low value in the dark (Fig. 3a; 73 ± 5%, Fig. 3b) but was observed to the same extent as that in the wild type under BL (Fig. 3a; 99 ± 2%, Fig. 3b). In contrast, no promotion of uptake by *Cr*PHOT under BL was observed in the *fpk1*Δ *fpk2*Δ *lem3*Δ background strain (Fig. 3a; 31 ± 9% in the dark and 32 ± 7% under BL for NBD-PE, Fig. 3b). This indicates that NBD-PE uptake promoted by *Cr*PHOT depends on Dnf1p/Dnf2p-Lem3p. The same results were obtained when NBD-PC was added (Fig. 3b, Supplementary Fig. S3). These results suggest that P4-ATPase-mediated phospholipid uptake can be photo-controlled via *Cr*PHOT. In addition, when the *fpk1*Δ *fpk2*Δ mutant harboured KDm plasmids, intracellular fluorescence was hardly observed even under BL (Fig. 3a; 50 ± 8% for NBD-PE and 64 ± 13% for NBD-PC under BL, Fig. 3b; Supplementary Fig. S3), suggesting that control by *Cr*PHOT is dependent on its own kinase activity.

These results were consistent with the altered sensitivity of the *fpk1*Δ *fpk2*Δ mutant expressing *Cr*PHOT or its derivatives to duramycin (Supplementary Fig. S4)^37^. Duramycin is a peptide toxin that specifically binds to PE in biological membranes^45,46^. The *fpk1*Δ *fpk2*Δ mutant is sensitive to duramycin because PE is not enriched in the inner leaflet and is exposed on the outer leaflet of the plasma membrane^32,34,39,43^. *Cr*PHOT suppressed the sensitivity of the *fpk1*Δ *fpk2*Δ mutant to duramycin in a BL-dependent manner, and the K-fragment suppressed it regardless of light conditions. In contrast, the *fpk1*Δ *fpk2*Δ mutant harbouring KDm showed increased sensitivity to duramycin even under BL (Supplementary Fig. S4)^37^. Under BL, the resistance activity by LOV1/2 m remained moderate, although it decreased the suppression compared to that of *Cr*PHOT (Supplementary Fig. S4). These results reconfirm the suggestion of controlling phospholipid flipping by light as described above.

We next investigated the time-course of phospholipids uptake in response to BL. Cells grown in the dark were further incubated for a total of 3 h. During incubation, cells were irradiated with different lengths of BL (0, 0.5, 1, 3 h) towards the end of the period. NBD-PE was added 1 h before the end of incubation time (Fig. 3c). As a result of quantification by flow cytometry, the internalization of NBD-PE reached the same level as that of the wild-type in 1 h BL irradiation (112 ± 7% relative to wild-type levels, Fig. 3c), although the effect of 0.5 h BL was not clear. These data demonstrate that this *Cr*PHOT system results in a superior, light-induced control of flipping activity.

### Light regulates both actin depolarization and switching of apical-isotropic growth

**Figure 4:**
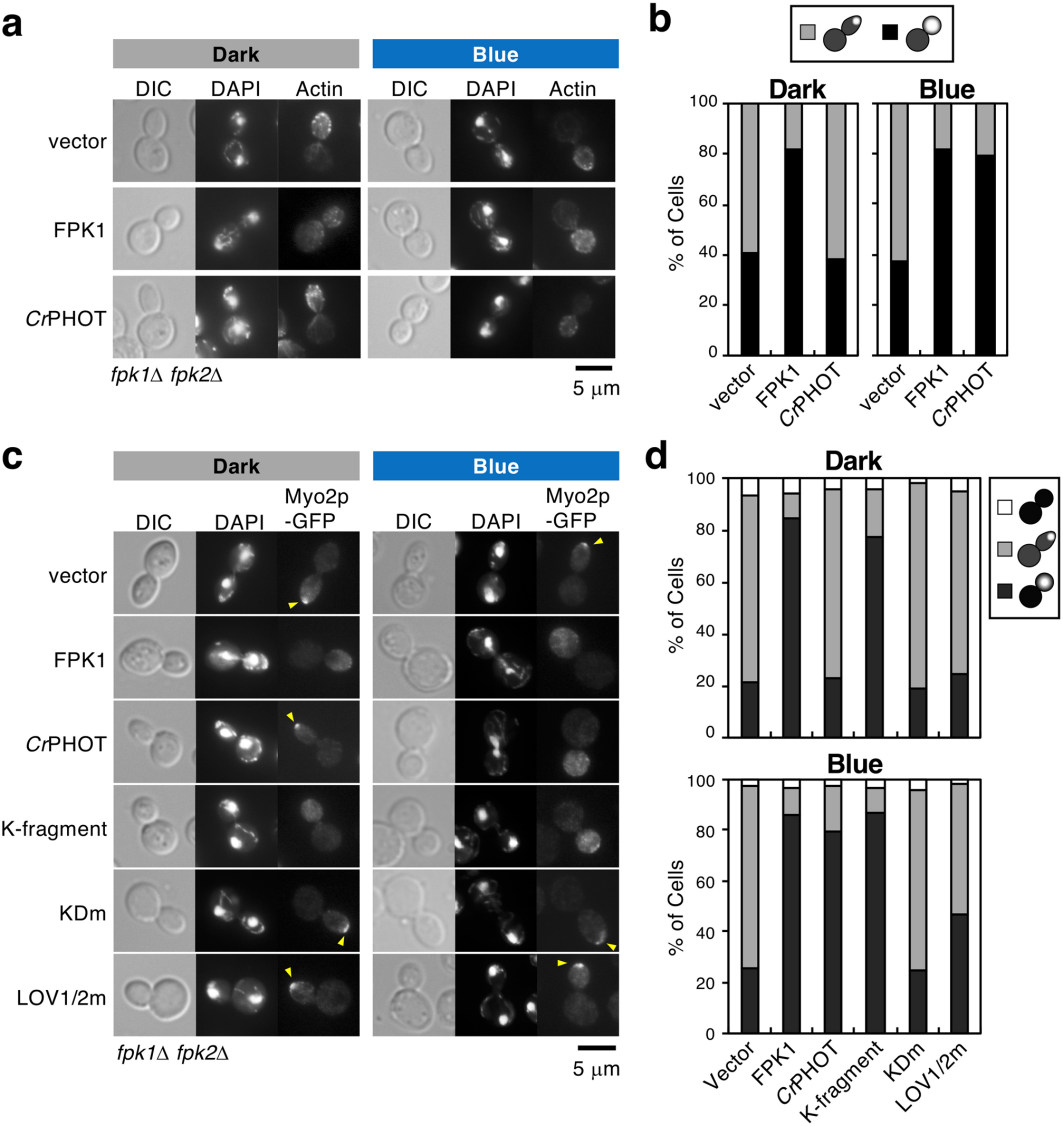
Optical control of actin depolarization associated with apical-isotropic growth switching. (**a**, **b**) Optical control of F-actin distribution by *Cr*PHOT. Yeast *fpk1*Δ *fpk2*Δ cells carrying pKT1639 (pRS416-FPK1) or pRS416-*Cr*PHOT were cultured in darkness (Dark) or under 10 μmol m^-2^ s^-1^ BL irradiation (Blue) at 18 °C, followed by staining with phalloidin-TRITC and DAPI to visualize actin and nuclei, respectively. (**a**) A representative cell image by microscopic observation. Scale bar = 5 μm. (**b**) Large-budded cells with divided nuclei were classified as showing actin polarized to the bud tip (grey) or its distribution in whole daughter cells (black). n = 155–253. (**c**, **d**) Optical control of Myo2p-GFP localization in a kinase-dependent manner. KKT353 (*fpk1*Δ *fpk2*Δ *MYO2-GFP*) cells carrying pKT1639 (pRS416-FPK1) or pRS416-*Cr*PHOT or its derivatives were cultured in YPDA medium in darkness (Dark) or under BL irradiation (Blue) at 18 °C, followed by staining with DAPI to visualize nuclei. (**c**) A representative cell image obtained by microscopic observation. Arrowheads indicate Myo2p-GFP polarized to the bud tip. Scale bar = 5 μm. (**d**) Large-budded cells with divided nuclei were classified as showing Myo2p-GFP distributed in whole daughter cells (black), polarized to the bud tip (grey) or delocalized (white). n = 96–180.

In small-budded cells (at G2/early mitotic phase), cortical actin patches (small assemblage of actin filaments) are polarized at the tip of the bud, and daughter cells exhibit apical growth^47^. In large-budded cells (at a late mitotic phase), the actin patches are randomly distributed, and the cells switch to isotropic growth^47^. Dnf1p/Dnf2p-Lem3p and Fpk1p/Fpk2p are involved in actin depolarization and switching from apical to isotropic growth. Therefore, defects in these genes result in prolonged polarization of actin patches at the tip and extended elongation of the bud even at the late mitotic phase^31,32^. We confirmed this phenotype in the *fpk1*Δ *fpk2*Δ mutant. Cells cultured in each light condition were stained with phalloidin-TRITC (tetramethylrhodamine B isothiocyanate peptide) to visualize actin and with DAPI (4′,6-diamidino-2-phenylindole) to confirm the cell cycle stage. As a result, the polarized actin patches and elongated bud shape were most prominent in large-budded cells regardless of light conditions (Fig. 4a, vector), and the ratio of cells with dispersed actin patches was much lower than that in FPK1p-expressing cells (Fig. 4a, vector; 40.8% in the dark and 37.5% under BL, Fig. 4b, vector). If light controls P4-ATPase activity, it will also restore both switching of actin positioning and proper growth direction. We next examined the localization of actin patches and bud morphology in the *fpk1*Δ *fpk2*Δ mutant expressing *Cr*PHOT. In the dark, a low number of cells had depolarized actin (Fig. 4a; 38.5%, Fig. 4b), and the bud morphology remained tapered in large-budded cells. When cells were irradiated with BL, actin patches were distributed throughout the daughter cells, and the buds exhibited a round shape similar to FPK1p-expressing cells (Fig. 4a; 79.3%, Fig. 4b). These results suggest that *Cr*PHOT is able to control both actin depolarization and switching of cell growth in a BL-dependent manner.

The same results were obtained in the localization analysis of Myo2p-GFP (Fig. 4c, d). Type V myosin Myo2p transports polarity proteins and is mostly localized at the bud tip during bud formation and dispersed throughout the cell at the late mitotic phase^48^. In the *lem3*Δ or the *fpk1*Δ *fpk2*Δ mutant, Myo2p-GFP remains polarized at the bud tip even at the late mitotic phase^31,32^. We thus investigated the photo-regulation of Myo2p-GFP localization by *Cr*PHOT. In the *fpk1*Δ *fpk2*Δ mutant expressing *Cr*PHOT, Myo2p-GFP was polarized in the dark, but it was distributed throughout the cells under BL as *FPK1* was (Fig. 4c; 23.0% in the dark and 79.6% under BL, ratio of cells with dispersed Myo2p-GFP, Fig. 4d). Depolarization of Myo2p-GFP was observed in the cells expressing the K-fragment regardless of light irradiation (77.2% in the dark and 86.7% under BL), while prolonged polarization was observed even under BL in KDm- (Fig. 4c; 19.3% in the dark and 25.0% under BL, Fig. 4d) or LOV1/2 m-expressing cells (Fig. 4c; 24.7% in the dark and 46.6% under BL; Fig. 4d). These results suggest that light can regulate cell polarity switching in a *Cr*PHOT kinase activity-dependent manner.

### Light regulates endocytic recycling to the TGN in the CrPHOT system

**Figure 5:**
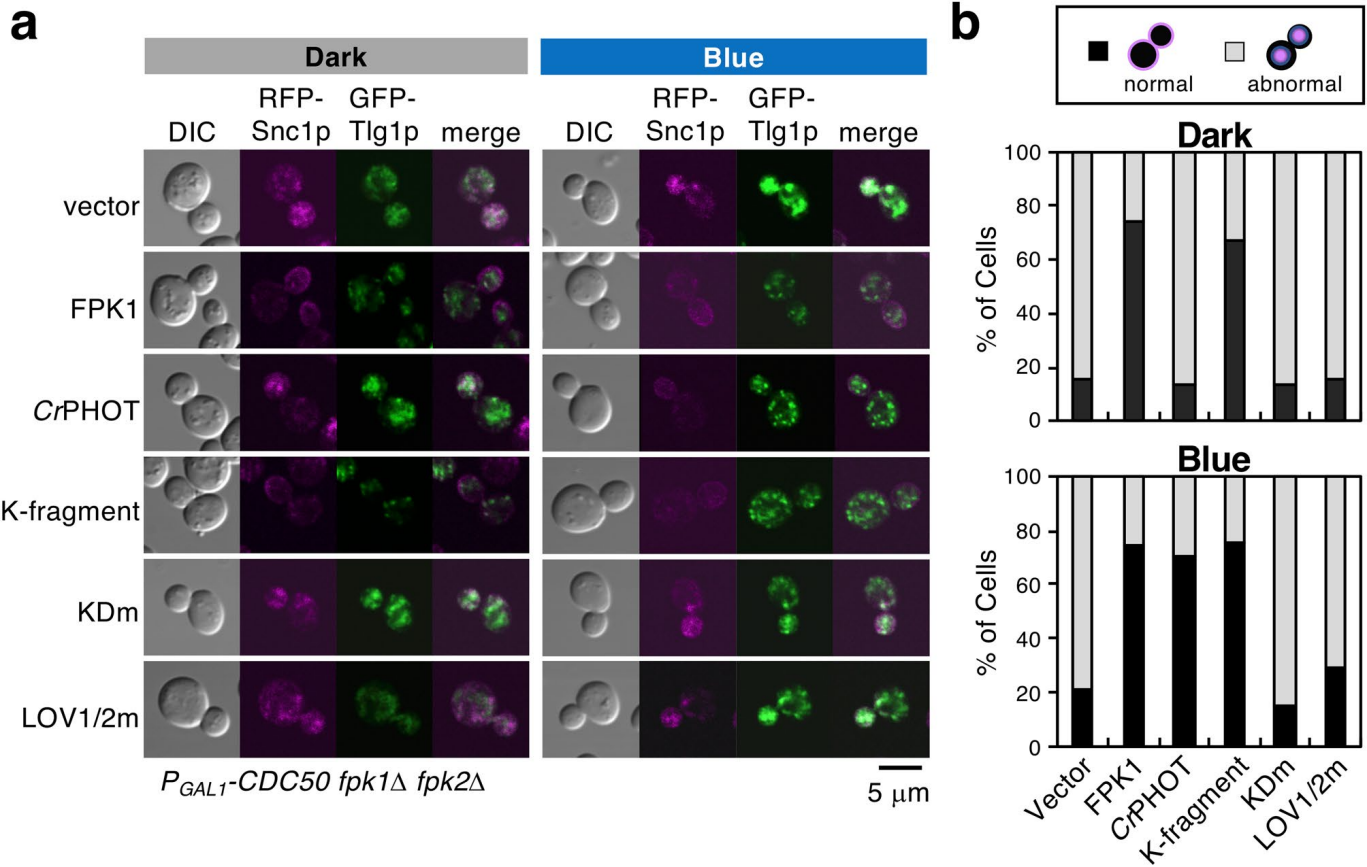
Optical control of endocytic recycling from early endosomes to the TGN. YTM2110 (*P*_*GAL1*_*-CDC50 fpk1*Δ *fpk2*Δ *mRFP-SNC1*) cells carrying pKT1651 (pRS315-GFP-TLG1) and pKT1639 (pRS416-FPK1) or pRS416-*Cr*PHOT or its derivatives were cultured in darkness (Dark) or under 10 μmol m^−2^ s^−1^ BL irradiation (Blue) at 18 °C for 12 h to deplete Cdc50p in YPDA medium, followed by fixation with 0.5% formaldehyde. (**a**) A representative cell image by confocal microscopic observation. Scale bar = 5 μm. (**b**) Cells were classified as showing mRFP-Snc1p localized in the plasma membrane (normal, black) or in abnormal intracellular structures (abnormal, grey). n = 155–315.

Dnf1p/Dnf2p-Lem3p and Fpk1p/Fpk2p, redundantly with Drs2p-Cdc50p, regulate endocytic recycling. The *lem3*Δ or *fpk1*Δ *fpk2*Δ mutant with Cdc50p-depletion exhibits severe defects in the retrieval pathway from early endosomes to the TGN^22,32,33^. We thus investigated whether *Cr*PHOT can regulate this pathway in a light-dependent manner. An exocytic vesicle-SNARE Snc1p is recycled from the plasma membrane via early endosomes to the TGN by this pathway^49^. mRFP-Snc1p is primarily localized at the plasma membrane of daughter cells during bud formation in partially Cdc50p-depleted *fpk2*Δ cells (Fig. 5a, FPK1)^32^. A target-SNARE, Tlg1p, is recycled between the TGN and early endosomes, and thus, Tlg1p-GFP was observed in punctate fluorescence, indicating endosome/TGN localization in this cell (Fig. 5a, FPK1)^50^. When the retrieval pathway from early endosomes to the TGN is inhibited, Snc1p and Tlg1p accumulate in abnormal structures in the cell (Fig. 5a, vector)^32,33^.

Plasmids encoding *Cr*PHOT or its derivatives were introduced into the Cdc50p-depleted *fpk1*Δ *fpk2*Δ mutant expressing mRFP-Snc1p and GFP-Tlg1p and cultured in the dark or under BL. After fixing the cells with formaldehyde, the localization of those marker proteins was observed using a microscope. In *Cr*PHOT-expressing cells, mRFP-Snc1p and GFP-Tlg1p were observed in the abnormal aggregates in the dark; cells in which mRFP-Snc1p was normally localized in the plasma membrane were hardly observed (Fig. 5a; 13.3%, Fig. 5b, *Cr*PHOT) as in the case of vector (15.0% in the dark, 20.9% under BL). Under BL irradiation, GFP-Tlg1p was localized in the normal punctate structures, and mRFP-Snc1p was localized in the cell periphery (Fig. 5a; 70.7%, Fig. 5b, *Cr*PHOT), as in the case of the cells expressing Fpk1p (Fig. 5a; 74.5% in the dark, 74.2% under BL, Fig. 5b, FPK1). LOV1/2 m exhibited abnormal aggregation of both marker proteins and failed to restore them to normal localization even under BL irradiation (Fig. 5a; 15.5% in the dark, 29.2% under BL, Fig. 5b, LOV1/2 m). These results suggest that the endocytic recycling pathway can be photo-controlled by *Cr*PHOT. We then investigated the necessity for kinase activity in the photo-control of endocytic recycling. As a result, both mRFP-Snc1p and GFP-Tlg1p showed normal localization in cells harbouring the K-fragment regardless of light irradiation (Fig. 5a; 67.1% in the dark and 75.0% under BL, Fig. 5b), but failed for KDm even under BL (Fig. 5a; 13.9% in the dark and 15.5% under BL, Fig. 5b). These results suggest that the control of endocytic recycling by light requires *Cr*PHOT kinase activity.

## Discussion

In this study, we succeeded in photo-controlling phospholipid (such as PC and PE) flipping and biological membrane functions (actin depolarization and endocytic recycling) in yeast. To date, some optogenetic techniques have been established to transiently control the amounts of phosphatidylinositol 4,5- bisphosphate (PI (4,5) P2) and its metabolites in cells using PI-metabolizing enzymes^51^. The tool we proposed is the first technique to optically control the intracellular distribution of non-PI phospholipids.

The basic mechanism of this new tool is that *Cr*PHOT light-controls the activity of select P4-ATPases in *S. cerevisiae*. The P4-APases localized in the plasma membrane of yeast are mainly Dnf1p/Dnf2p-Lem3p^22,23,24,25^. In the NBD-phospholipid uptake analysis, NBD-PC and NBD-PE added to the medium were taken up into the cell in a light-dependent manner in the presence of *Cr*PHOT, and the *lem3*Δ mutation impaired this uptake (Fig. 3). These results provide direct evidence that *Cr*PHOT optically regulates the flipping of PE and PC through activation of Dnf1p/Dnf2p-Lem3p in the plasma membrane. Furthermore, we showed that NBD-PE uptake by *Cr*PHOT reached that of the wild-type cells at least within one hour of BL irradiation (Fig. 3c). Therefore, the activation itself of flippase by BL is presumed to occur in a short time. However, the *fpk1*Δ *fpk2*Δ mutant harbouring *Cr*PHOT plasmids showed slightly higher NBD-phospholipids uptake even in the dark (Fig. 3b,c). In the case of KDm, the uptake activity was suppressed to the same level as that of empty vector (Fig. 3b), hence the *Cr*PHOT activity within yeast cells is presumed to leak in the dark. Improvements of *Cr*PHOT molecules to strictly inhibit activity in the dark are awaited. Although detailed analysis of photo-reversibility is also needed in the future, we provide the first basis of a tool for optic controlling lipid uptake in this study.

How does *Cr*PHOT regulate P4-ATPases? All P4-ATPase-related responses analysed in this study were dependent on the kinase activity of *Cr*PHOT (Figs. 1, 3, 4, 5, Supplementary Fig. S3 and S4). Yeast Fpk1p phosphorylates Dnf1p/Dnf2p P4-ATPases in vivo and in vitro^32,35,36^. The kinase activity of Fpk1p and phosphorylation of Dnf1p are required for the optimal function of Dnf1p^32,35^. The control of P4-ATPase activity by Fpk1p/Fpk2p and control of P4-ATPase activity by *Cr*PHOT under BL irradiation were remarkably consistent; therefore, we conclude that *Cr*PHOT also regulates Dnf1p/Dnf2p by phosphorylation. Some phosphorylation sites of Dnf1p by Fpk1p have been identified, and the combination of mutations at those sites within Dnf1p reduces PE flipping in yeast cells^35^. The molecular mechanism underlying P4-ATPase regulation by kinase-related phosphorylation is still unknown, but this system that switches the activity of P4-ATPases by light will be quite useful for elucidating it in the future.

Next, it is necessary to consider the effect of *Cr*PHOT on factors other than Dnf1p/Dnf2p, e.g., other P4-ATPases. In microscopic observation, localization of *Cr*PHOT to TGN/endosomes was mainly observed (Fig. 2, Supplementary Fig. S2). Drs2p and Dnf3p are P4-ATPases mainly localized to the TGN/endosomes. Furthermore, their function in the endocytic recycling pathway is partially redundant with that of Dnf1p/Dnf2p^22,23,24,33^. Drs2p and Dnf3p are also phosphorylated by Fpk1p in vitro^32,36^, and Dnf3p is isolated as a phosphorylation substrate for Fpk1p/Fpk2p in vivo^34,35^. However, the necessity of Fpk1p/Fpk2p activity to the functions of Drs2p and Dnf3p is currently unknown. Further analysis is needed to determine whether *Cr*PHOT regulates the functions of other P4-ATPases, such as Drs2p and Dnf3p.

Another is the effects of Fpk1p/Fpk2p on factors other than P4-ATPases. The protein kinases Ypk1p and Akl1p are known as phosphorylation substrates for Fpk1^34,35,36,43^. Target of rapamycin complex 2 (TORC2) serves as a sensor and regulator for plasma membrane status and is involved in actin-cytoskeleton regulation, sphingolipid synthesis, and endocytosis in *S. cerevisiae*^52,53^. Under plasma membrane stresses, TORC2 phosphorylates and negatively regulates Fpk1p through activation of Ypk1p^34^. Fpk1p activated under normal conditions suppresses the upstream inhibitor Ypk1p by phosphorylation and promotes the endocytic pathway from the plasma membrane through inhibition of Akl1p by phosphorylation, independent of Dnf1p/Dnf2p activation^35,36^.

To investigate the involvement of *Cr*PHOT in this endocytic pathway, *CrPHOT* or *FPK1* was introduced into the *P*_*GAL1*_*-CDC50 fpk1*Δ *fpk2*Δ strain, and endocytosis from the plasma membrane was observed by FM4-64 staining. Unfortunately, the *P*_*GAL1*_*-CDC50 fpk1*Δ *fpk2*Δ cells failed to show any FM4-64 uptake delay even when the corresponding empty vector was introduced, despite experiments at various temperatures (4–24 °C) and depression conditions (data not shown). This observation indicated that the endocytic pathway functioned properly even in the absence of Dnf1p/Dnf2p-Lem3p and Drs2p-Cdc50p in our experimental conditions. Therefore, the involvement of *Cr*PHOT in endocytosis could not be investigated. We would need to investigate whether *Cr*PHOT regulates this pathway, including the phosphorylation of Akl1p and Ypk1p.

RSK3 (belonging to the p90-S6K subfamily) and Ca^2+^-dependent protein kinase C (PKC) are known as kinases that would be involved in phosphorylation of P4-ATPases in mammals. RSK3 has been identified as a functional counterpart of Fpk1p/Fpk2p in yeast screening^43^, but P4-ATPase regulation and phosphorylation in mammalian cells are not understood. PKC controls the endocytosis of the P4-ATPase ATP11C (relates to B-cell maturation, erythrocyte shape, anaemia and hyperbilirubinemia), and phosphorylation of the ATP11C C-terminal region is required for endocytosis^54^. Hence, P4-ATPases are likely regulated by kinases in many eukaryotes, and *Cr*PHOT may also function in mammalian cells. Although an analysis of photo-reversibility and improvement in controllability are required, this study is able to propose the basis of a P4-ATPase light control system.

## Methods

### Media and growth conditions

Yeast strains were cultured in YPDA-rich medium (1% yeast extract [BD Biosciences, San Jose, CA], 2% bacto-peptone [BD], 2% glucose [Nacalai Tesque, Kyoto, Japan], and 0.01% adenine [FUJIFILM Wako Pure Chemical Corporation, Osaka, Japan]). Strains carrying plasmids were selected in synthetic medium (SD) containing the required nutritional supplements^55^. When appropriate, 0.5% casamino acids [BD] were added to SD medium without uracil [FUJIFILM Wako] (SDA-Ura). For induction of the *GAL1* promoter, 3% galactose [FUJIFILM Wako] and 0.2% sucrose [Nacalai] were used as carbon sources instead of glucose (YPGA and SGA-Ura). Blue (peak at 470 nm) and red (peak at 660 nm) light-emitting diode panels [SL-150X150 series; CCS, Tokyo, Japan] were used as light sources. In all analyses, BL and RL were used at an intensity of 10 μmol m^−2^ s^−1^ unless otherwise noted. When grown on the agar medium, the yeast cells were irradiated with light vertically from a height of about 10 cm above the plate. When cultured in a liquid medium, the entire test tube was irradiated with light from a distance of about 10 cm from the side of the tube. *Escherichia coli* strains were cultured in LB medium [Nacalai] containing appropriate antibiotics as needed. The lithium acetate method was used to introduce plasmids into yeast cells^56,57^.

### Strains and plasmids

The *S. cerevisiae* strains used in this study are listed in Table 1. Yeast strains carrying monomeric red fluorescent protein (mRFP)-tagged *SNC1* were constructed by integrating linearized pRS306-mRFP-SNC1 into the *URA3* locus, followed by a marker change from *URA3* to *TRP1*. Strain carrying SEC7-mRFP was constructed by PCR-based procedures as described^58,59^. *E. coli* strain DH5α was used for the construction and amplification of plasmids.

**Table 1:** Yeast strains used in this study.

Strain^a^	Relevant genotype	Derivation/source
BY4743	*MAT*a/α *LYS2/lys2*Δ*0 ura3*Δ*0/ura3*Δ*0 his3*Δ*1/his3*Δ*1 leu2*Δ*0/leu2*Δ*0 met15*Δ*0/MET15*	Brachmann et al., 1998^63^
YEF473	*MAT*a/α *lys2-810/ lys2-810 ura3-52/tura3-52 his3*Δ*-200/his3*Δ*-200 trp1*Δ*-63/trp1*Δ*-63 leu2*Δ*-1/leu2*Δ*-1*	Bi and Pringle, 1996^64^
KKT330	*MAT*a *LYS2 ura3*Δ*0 his3*Δ*1 leu2*Δ*0 MET15 HIS3MX6::P*_*GAL1*_*-3HA-CDC50 fpk1*Δ*::HphMX4 fpk2*Δ*::KanMX6* (designated here as *P*_*GAL1*_*-CDC50 fpk1*Δ *fpk2*Δ)	Nakano et al., 2008^32^
KKT268	*MAT*a *LYS2 ura3*Δ*0 his3*Δ*1 leu2*Δ*0 MET15 fpk1*Δ*::HphMX4 fpk2*Δ*::KanMX6* (designated here as *fpk1*Δ *fpk2*Δ)	Nakano et al., 2008^32^
KKT353	*MAT*a *LYS2 ura3*Δ*0 his3*Δ*1 leu2*Δ*0 MET15 MYO2-GFP::HIS3MX6 fpk1*Δ*::HphMX4 fpk2*Δ*::KanMX6*	Nakano et al., 2008^32^
YKT905	*MAT*a *ura3-52 his3*Δ*-200 trp1*Δ*-63 leu2*Δ*-1 lys2-801 SEC7-mRFP::TRP1*	Sakane et al., 2006^65^
KKT492	*MAT*a *LYS2 ura3*Δ*0 his3*Δ*1 leu2*Δ*0 MET15 HIS3MX6::P*_*GAL1*_*-3HA-CDC50 fpk1*Δ*::HphMX4 fpk2*Δ*::KanMX6 ura3::TRP1::mRFP-SNC1*	This study
KKT332	*MAT*a *lys2*Δ*0 ura3*Δ*0 his3*Δ*1 leu2*Δ*0 met15*Δ*0 DNF1-GFP::HIS3MX6 fpk1*Δ*::HphMX4 fpk2*Δ*::KanMX6*	Nakano et al., 2008^32^
KKT336	*MAT*a *LYS2 ura3*Δ*0 his3*Δ*1 leu2*Δ*0 MET15 DNF2-GFP::HIS3MX6 fpk1*Δ*::HphMX4 fpk2*Δ*::KanMX6*	Nakano et al., 2008^32^
KKT274	*MAT*a *LYS2 ura3*Δ*0 his3*Δ*1 leu2*Δ*0 MET15 lem3*Δ*::KanMX6 fpk1*Δ*::HphMX4 fpk2*Δ*::HIS3MX6*	This study

^a^KKT strains are isogenic derivatives of BY4743. YKT strains are isogenic derivatives of YEF473.

The plasmids used in this study are listed in Table 2. pRS416-GFP-*Cr*PHOT, pRS416-mRFP-*Cr*PHOT, pRS416-GFP-KDm and pRS416-mRFP-KDm were constructed as follows. GFP- or mRFP-tagged *CrPHOT* or *CrPHOT(D549N)*^37^ was constructed by megaprimer PCR-based procedures^60^ and cloned into the *Bam*HI/*Sal*I site of pRS416^61^. pRS416-LOV1/2 m was generated using a QuikChange site-directed mutagenesis kit [Agilent Technologies, Santa Clara, CA] with pRS416-LOV2m^37^. The genes inserted into pRS416 were constitutively expressed in a form tagged with two N-terminal tandem repeats of the influenza virus haemagglutinin epitope (2HA) under control of the *TPI1* promoter. All regions constructed by PCR-based procedures were verified by DNA sequencing.

**Table 2:** Plasmids used in this study.

Plasmid	Characteristics	Derivation/source
YCplac111	*LEU2 CEN4*	Gietz and Sugino, 1988^66^
YEplac181	*LEU2 2 μm*	Gietz and Sugino, 1988^66^
YEplac195	*URA3 2 μm*	Gietz and Sugino, 1988^66^
pKO10	*P*_*GAL1*_*-HA URA3 2 μm*	Kikyo et al., 1999^67^
pRS416	*URA3 CEN6*	Sikorski and Hieter, 1989^61^
pRS315	*LEU2 CEN6*	Sikorski and Hieter, 1989^61^
pKT1634 [pRS416-GFP-FPK1]	*P*_*TPI1*_*-GFP-FPK1 URA3 CEN6*	Nakano et al., 2008^32^
pKT1638 [pRS416-mRFP-FPK1]	*P*_*TPI1*_*-mRFP-FPK1 URA3 CEN6*	Nakano et al., 2008^32^
pKT1639 [pRS416-HA-FPK1]	*P*_*TPI1*_*-HA-FPK1 URA3 CEN6*	Nakano et al., 2008^32^
pKT1651 [pRS315-GFP-TLG1]	*P*_*TPI1*_*-GFP-TLG1 LEU2 CEN6*	This study
pKT2177 [pRS306-mRFP-SNC1] pRS416-CrPHOT	*P*_*TPI1*_*-mRFP-SNC1 URA3* *P*_*TPI1*_*-HA-CrPHOT URA3 CEN6*	This study Aihara et al., 2012^37^
pRS416-K-fragment	*P*_*TPI1*_*-HA-CrPHOT*Δ*N URA3 CEN6* (identical to Kinase-fragment in Aihara et al., 2012^37^)	Aihara et al., 2012^37^
pRS416-KDm	*P*_*TPI1*_*-HA-CrPHOT(D549N) URA3 CEN6* (identical to Kinase-dead (D549N) in Aihara et al., 2012^37^)	Aihara et al., 2012^37^
pRS416-LOV2m	*P*_*TPI1*_*-HA-CrPHOT(C250A) URA3 CEN6* (identical to (C250A) in Aihara et al., 2012^37^)	Aihara et al., 2012^37^
pRS416-LOV1/2 m	*P*_*TPI1*_*-HA-CrPHOT(C57A, C250A) URA3 CEN6*	This study
pRS416-GFP-CrPHOT	*P*_*TPI1*_*-HA-GFP-CrPHOT URA3 CEN6*	This study
pRS416-mRFP-CrPHOT	*P*_*TPI1*_*-HA-mRFP-CrPHOT URA3 CEN6*	This study
pRS416-GFP-KDm	*P*_*TPI1*_*-HA-GFP-CrPHOT*Δ*N URA3 CEN6*	This study
pRS416-mRFP-KDm	*P*_*TPI1*_*-HA-mRFP-CrPHOT*Δ*N URA3 CEN6*	This study

### Yeast growth assay

The yeast cells were grown at 28 °C in liquid SGA-Ura medium to an A_600_ of 0.6–0.8 and diluted with sterile water to an A_600_ of 0.1. Ten-fold serial dilutions (10^–1^, 10^–2^, 10^–3^, 10^–4^) were made in sterile water. A 10-μl aliquot of each of the diluted cell suspensions was then spotted on a plate with SGA-Ura or SDA-Ura medium. The plates were then placed at 28 °C under different light conditions for 3 days. For growth sensitivity to duramycin, the yeast cells grown in liquid SDA-Ura were diluted as above, and 2 μl of each of the diluted cell suspensions were examined on YPDA plates containing 20 μM duramycin [Sigma-Aldrich, St. Louis, MO] at 28 °C.

### Immunoblot analysis

Yeast cells were grown to logarithmic phase in YPGA medium at 28 °C under different light conditions. Protein extraction from yeast cells was performed as described^62^. The extracted proteins (40 μg) were separated in a 4–15% SDS-polyacrylamide gel [Bio-Rad Laboratories, Hercules, CA] and blotted onto polyvinylidene difluoride (PVDF) membrane [Bio-Rad], and immunoblotting was performed using mouse anti-HA monoclonal antibody [MBL, Nagoya, Japan] and horseradish peroxidase (HRP)-conjugated anti-mouse IgG [Promega, Madison, WI]. The signals were detected using an LAS-3000 imaging system [FUJIFILM, Tokyo, Japan]. The raw image is presented in Supplementary Fig. S5, which includes an unprocessed original data that was used to prepare Fig. 1d.

### Microscopic observations

Microscopic observations were performed as previously described^32^. Yeast cells were grown to early-midlogarithmic phase in YPDA liquid medium at 18 °C in darkness or under BL irradiation. All experiments requiring dark conditions were performed in a dark room with the aid of green safe light. Most GFP- or mRFP-tagged proteins were observed in living cells, which were observed using a Fluoview FV1000 confocal microscope [Olympus Corporation, Tokyo, Japan]. The lipophilic styryl dye FM4-64 [Thermo Fisher Scientific, Waltham, MA, USA] was used to visualize endosomal structures. Cells grown in the logarithmic phase were harvested by centrifugation, washed twice with ice-cold SC medium, resuspended in 100 μl of ice-cold SC medium containing 4 μl of 1 mM FM4-64 in dimethyl sulfoxide (DMSO) (40 μM final concentration), and then incubated at 25 °C for 20 min. Labelled cells were immediately observed using a confocal microscope. Localization of mRFP-Snc1p and GFP-Tlg1p or Sec7p-mRFP was observed in fixed cells. Cells grown in the logarithmic phase were fixed by the addition of formaldehyde (3.7% final concentration) [FUJIFILM Wako] into the medium and incubated at 18 °C for 10 min and then 30 °C for 10 min. After fixation, cells were washed twice with phosphate-buffered saline (PBS) and immediately observed using a confocal microscope. To observe Myo2p-GFP in cells with divided nuclei, cells fixed as above were stained with DAPI (0.5 μg ml^-1^ final concentration) [Sigma-Aldrich] in 100 μl of water at room temperature for 10 min. To visualize F-actin, cells were fixed by the addition of formaldehyde (5.0% final concentration) into the medium and incubated at 18 °C for 10 min and then 25 °C for 30 min. After fixation, the cells were labelled with phalloidin-TRITC (0.1925 μM final concentration) [Sigma-Aldrich] in PBS at room temperature for 30 min, washed three times with PBS, and then stained with DAPI as described above. Cells stained with DAPI were washed three times with water and immediately observed using a BX51 biological microscope [Olympus] with the appropriate fluorescence filter sets [Olympus]. Images were acquired with an ORCA-fusion digital CMOS camera [C14440-20UP; Hamamatsu Photonics, Hamamatsu, Japan].

### Internalization of fluorescence-labelled phospholipids into yeast cells

Large unilamellar vesicles containing NBD-phospholipids were prepared as described^29^. 1-palmitoyl-2-(6-NBD-aminocaproyl)-PE (NBD-PE), 1-palmitoyl-2-(6-NBD-aminocaproyl)-PC (NBD-PC), and dioleoylphosphatidylcholine (DOPC) were obtained from Avanti Polar Lipids (Alabaster, AL). Fluorescently labelled phospholipid internalization experiments were performed as described^29,30^. Briefly, cells were grown to early logarithmic phase in SDA-U medium at 30 °C in the dark. After dilution to 0.35 A600 ml^-1^, cells were incubated for 60 min at 30 °C with liposomes containing 40% NBD-phospholipid and 60% DOPC at a final concentration of 20 μM in the dark or under BL. Cells were then suspended in cold SD containing 20 mM sodium azide and 2.5% bovine serum albumin (BSA), incubated for 20 min, and washed with PBS. Flow cytometry of fluorescently labelled cells was performed on a FACSCanto II cytometer [BD]. For investigation of time response, overnight cultures were diluted to 0.2 A600 ml^-1^ and incubated for a total of 3 h in the indicated condition (dark, BL 0.5 h, BL 1.0 h, or BL 3.0 h), in which the last 1 h was incubated with the NBD-PE liposome. The experiments were performed in three biological replicates consisting of 10,000 cells per sample. Significance for Fig. 3 was determined using a one-way analysis of variance with a Tukey’s test.

## Supplementary information

Supplementary figures.
