# Supplementary material for: An optogenetic system to control membrane phospholipid asymmetry through flippase activation in budding yeast
Source: Sci Rep. 2020 Jul 27;10:12474. doi: 10.1038/s41598-020-69459-0 (PMC7385178; doi:10.1038/s41598-020-69459-0)
Supplement: Supplementary file 1 — Supplementary figures. [file 41598_2020_69459_MOESM1_ESM.pdf]

Supplemental Information

An Optogenetic System to Control Membrane Phospholipid Asymmetry through Flippases activation in Budding Yeast.

Authors:

Tomomi Suzuki[1,2,*], Tetsuo Mioka[3], Kazuma Tanaka[3] and Akira Nagatani[1,*]

Author affiliations:

[1]Department of Botany, Graduate School of Science, Kyoto University, Kyoto 606-8502, Japan

[2]PRESTO, Japan Science and Technology Agency, Kawaguchi, Saitama 332-0012, Japan

[3]Division of Molecular Interaction, Institute for Genetic Medicine, Hokkaido University Graduate School of Life Science, Kita-ku, Sapporo 060-0815, Japan

*Corresponding author:

Contact email: tomo@physiol.bot.kyoto-u.ac.jp and nagatani@physiol.bot.kyoto-u.ac.jp

Supplementary Figures

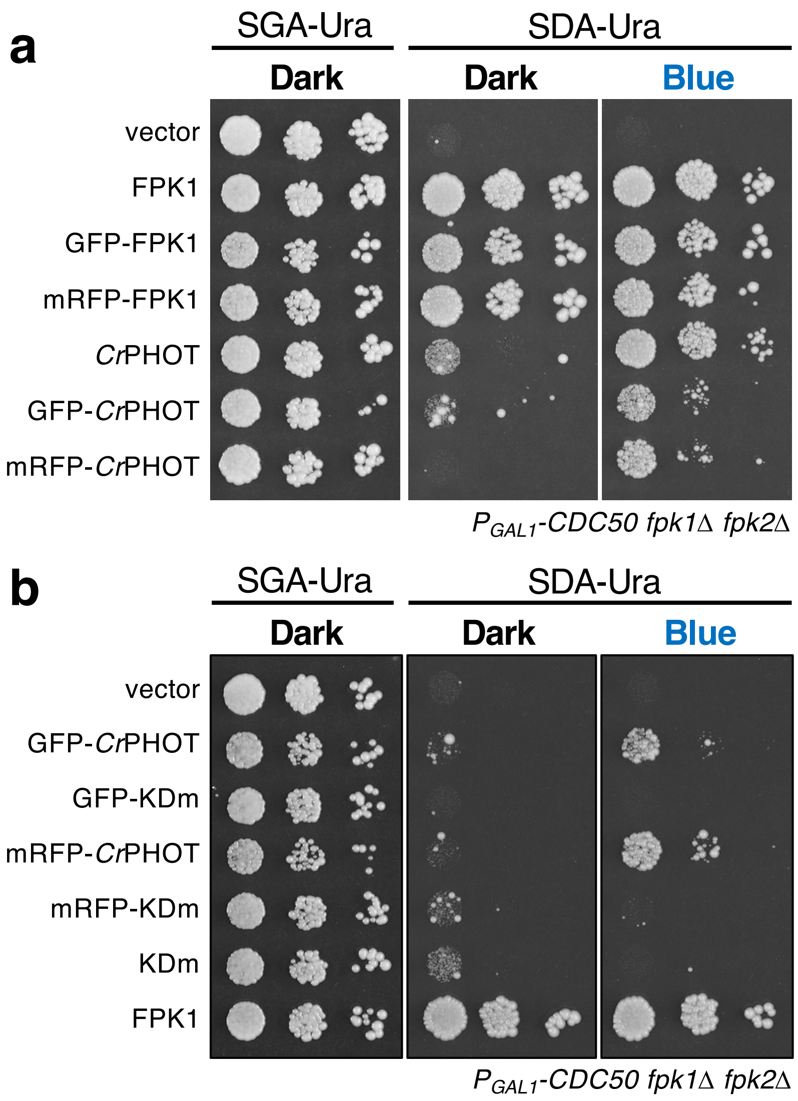

Supplementary Figure S1. The fluorescent-protein tagged *Cr*phot and KDm are also functional. Growth of the yeast conditional mutant P_{GAL1}-*CDC50 fpk1Δ fpk2Δ* expressing GFP or mRFP tagged *Cr*PHOT (a) and KDm, kinase-dead mutant with D549N (b). Yeast cells were serially diluted and spotted onto plates containing galactose (SGA-Ura) or glucose (SDA-Ura), which were incubated in darkness (Dark), or under 10 μmol m^{-2} s^{-1} BL irradiation (Blue) at 28 ˚C for 3 days.

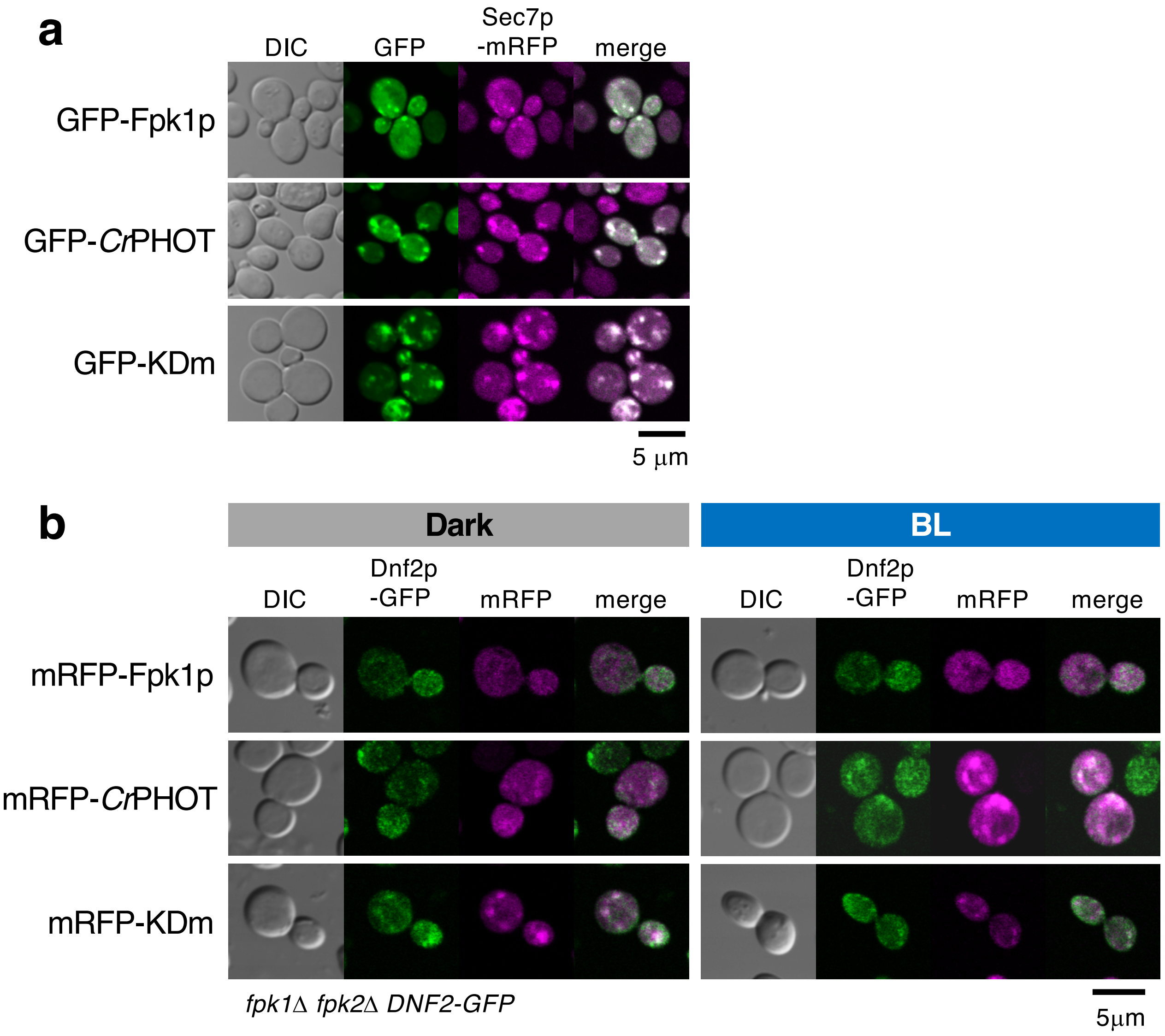

Supplementary Figure S2. Colocalization of *Cr*PHOT with Dnf2p or Sec7p. a Confocal images of YKT905 (*SEC7-mRFP*) cells expressing GFP tagged Fpk1p, *Cr*PHOT and KDm proteins under room light. **b** Confocal images of KKT336 (*fpk1Δ fpk2Δ*, *DNF2-GFP*) cells expressing mRFP tagged Fpk1p, *Cr*PHOT and KDm proteins. Yeast cells cultured in darkness (Dark) or under BL irradiation (Blue). Images merged to compare the two signal patterns. Scale bars = 5 μm.

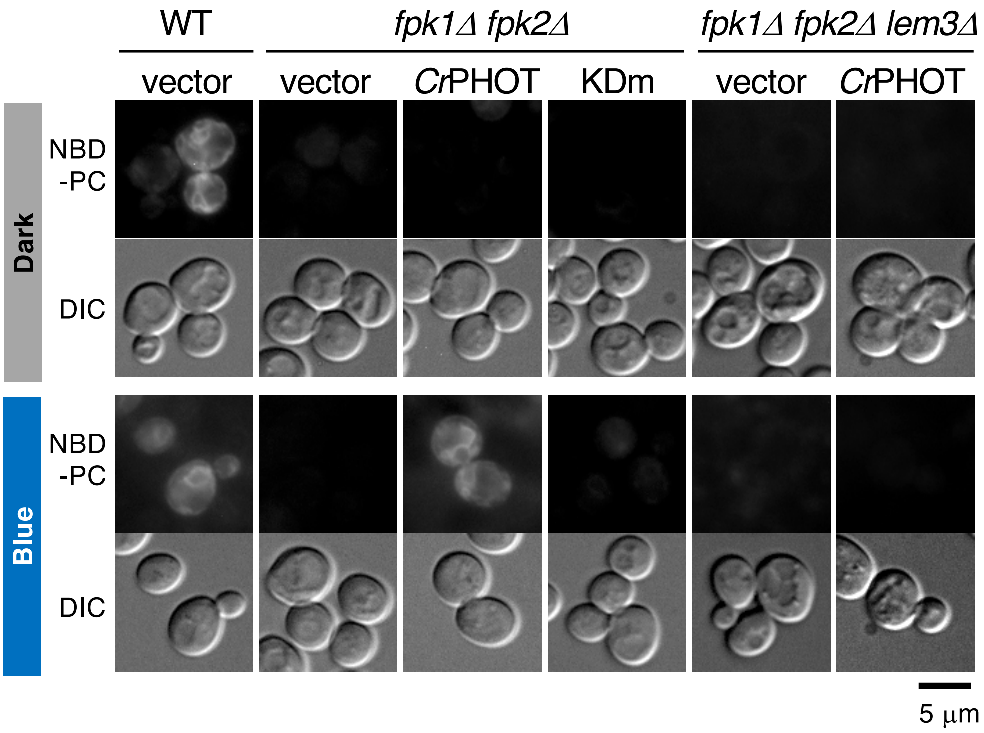

Supplementary Figure S3. NBD-PC internalization by *Cr*PHOT under BL. Wild-type (WT) harbouring vector plasmids, *fpk1*Δ *fpk2*Δ mutant harboring vector, *Cr*PHOT or KDm plasmids, and KKT274 (*fpk1*Δ *fpk2*Δ *lem3*Δ) harboring vector or *Cr*PHOT plasmids were grown in SC medium in the dark (Dark) or under BL (Blue) at 30 ˚C, and treated with NBD-PC. A representative cell image by confocal microscopic observation in each condition is shown. Scale bar = 5 μm.

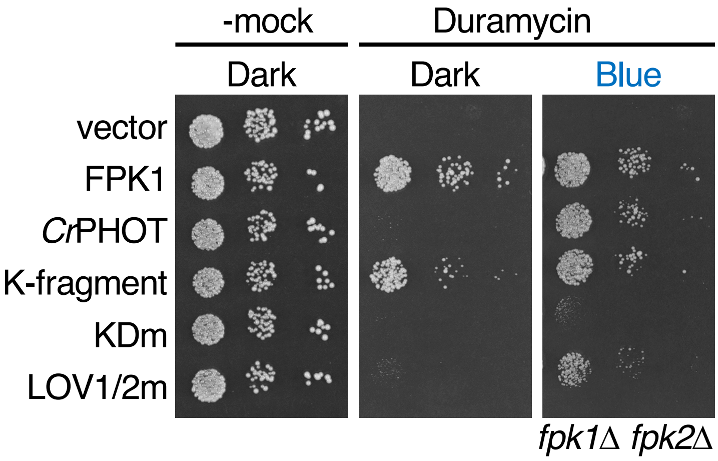

Supplementary Figure S4. Optical control of PE amount in the outer leaflet of the plasma membrane in a kinase-dependent-manner. Growth sensitivity of *fpk1*Δ *fpk2*Δ cells carrying pRS416-CrPHOT or its derivatives to PE-specific binding antibiotics, duramycin. Yeast cells were cultured in YPDA medium at 28 ˚C, serially diluted and spotted on to YPDA plate with or without 20 μM duramycin, which were incubated in darkness (Dark), or under BL irradiation (Blue) 28 ˚C for 2 days.

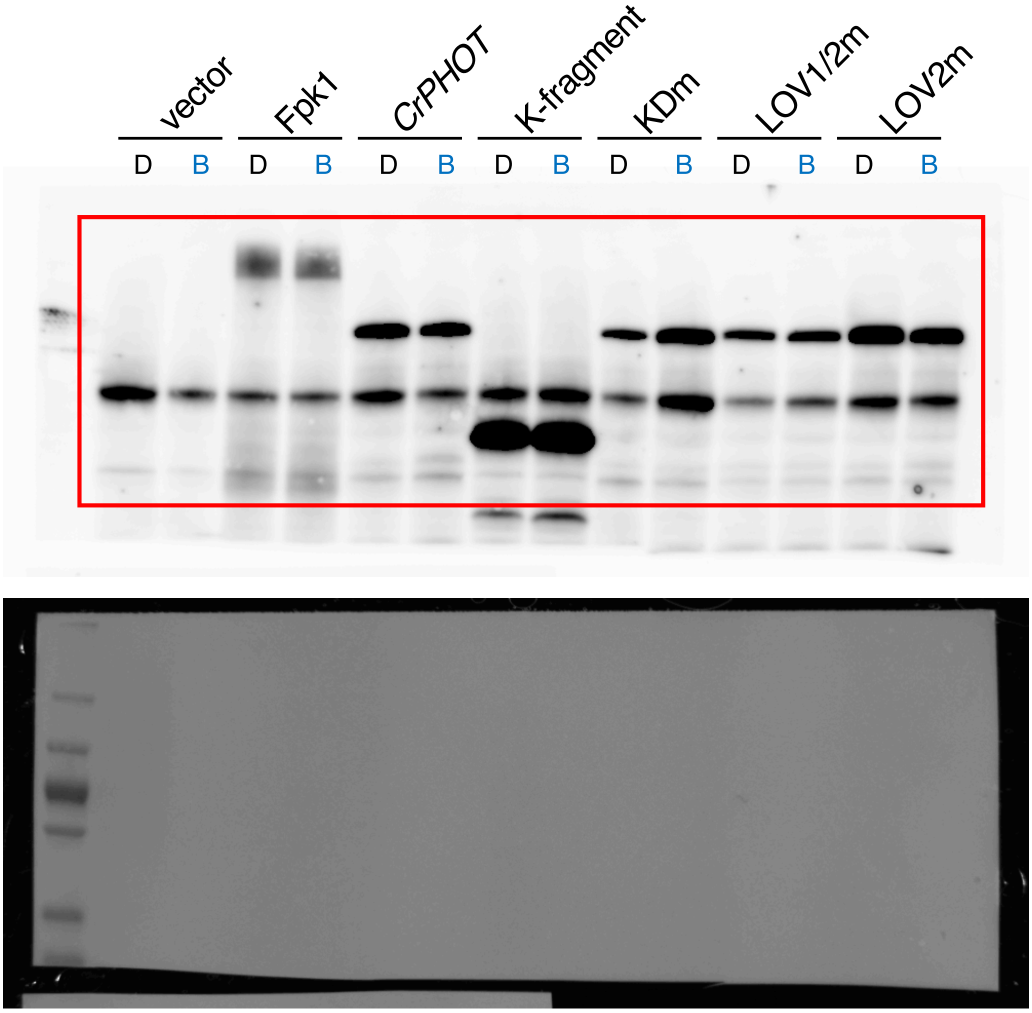

Supplementary Figure S5. The original unprocessed images of Western blots for Figure 1. Raw data of Western blots (upper panel) with corresponding membrane (lower panel). The area used in Fig. 1 is indicated by a red frame.
